# Diagnosis of cytomegalovirus corneal endotheliitis using surgically removed Descemet’s membrane and endothelium despite negative results with aqueous humor PCR: a case report

**DOI:** 10.1186/s12886-021-01962-y

**Published:** 2021-05-01

**Authors:** Suguru Nakagawa, Hitoha Ishii, Mitsuko Takamoto, Toshikatsu Kaburaki, Kiyoshi Ishii, Takashi Miyai

**Affiliations:** 1 Department of Ophthalmology, Saitama Red Cross Hospital, Saitama, Japan; 2 Department of Ophthalmology, University of Tokyo Graduate School of Medicine, 7-3-1 Hongo, Bunkyo-ku, Tokyo, Japan

**Keywords:** Cytomegalovirus, Corneal endotheliitis, Polymerase chain reaction, Ganciclovir, Descemet stripping automated endothelial keratoplasty

## Abstract

**Background:**

Cytomegalovirus (CMV) has been known to cause unilateral corneal endotheliitis with keratic precipitates and localized corneal edema, iridocyclitis, and secondary glaucoma. CMV endotheliitis is diagnosed based on clinical manifestations and viral examination using qualitative polymerase chain reaction (PCR) of the aqueous humor.

**Case presentation:**

An 80-year-old woman was referred to our department for bullous keratopathy. Pigmented keratic precipitates were found in the right eye without significant anterior chamber inflammation. After 8 months there was inflammation relapse with mutton fat keratic precipitates and PCR on aqueous humor was performed, with negative results for CMV, herpes simplex virus, and varicella zoster virus. Keratic precipitates disappeared with steroid instillation, and Descemet-stripping automated endothelial keratoplasty (DSAEK) was performed for the right eye. CMV-DNA was positive at 6.0 × 10^2^ copies/ GAPDH 10^5^ copies in real time PCR of corneal endothelial specimen removed during DSAEK with negative results for all the other human herpes viruses. After diagnosis of CMV corneal endotheliitis, treatment with systemic and topical ganciclovir was initiated and there was resolution of symptoms. No recurrence of iridocyclitis or corneal endotheliitis was observed at 6 months follow up.

**Conclusions:**

This case report suggests that PCR should be performed using the endothelium removed during DSAEK for bullous keratopathy of an unknown cause, even if PCR for aqueous humor yields negative results.

## Background

Cytomegalovirus (CMV) has been known to cause unilateral corneal endotheliitis with keratic precipitates (KPs) and localized corneal edema, iridocyclitis, and secondary glaucoma [1]. CMV endotheliitis is diagnosed based on clinical manifestations and viral examination using qualitative polymerase chain reaction (PCR) of the aqueous humor [2]. Patients are treated with the systemic and/or topical antiviral drug: ganciclovir [3]. We present a case of bullous keratopathy diagnosed as CMV endotheliitis based on the detection of CMV-DNA by PCR not in the aqueous humor but in the corneal endothelial specimen removed during Descemet-stripping automated endothelial keratoplasty (DSAEK).

## Case presentation

This retrospective case report was approved by the research ethics committee of the Graduate School of Medicine and Faculty of Medicine at the University of Tokyo and all procedures conducted in this study adhered to the tenets of the Declaration of Helsinki. Written informed consent was obtained.

**Fig. 1:**
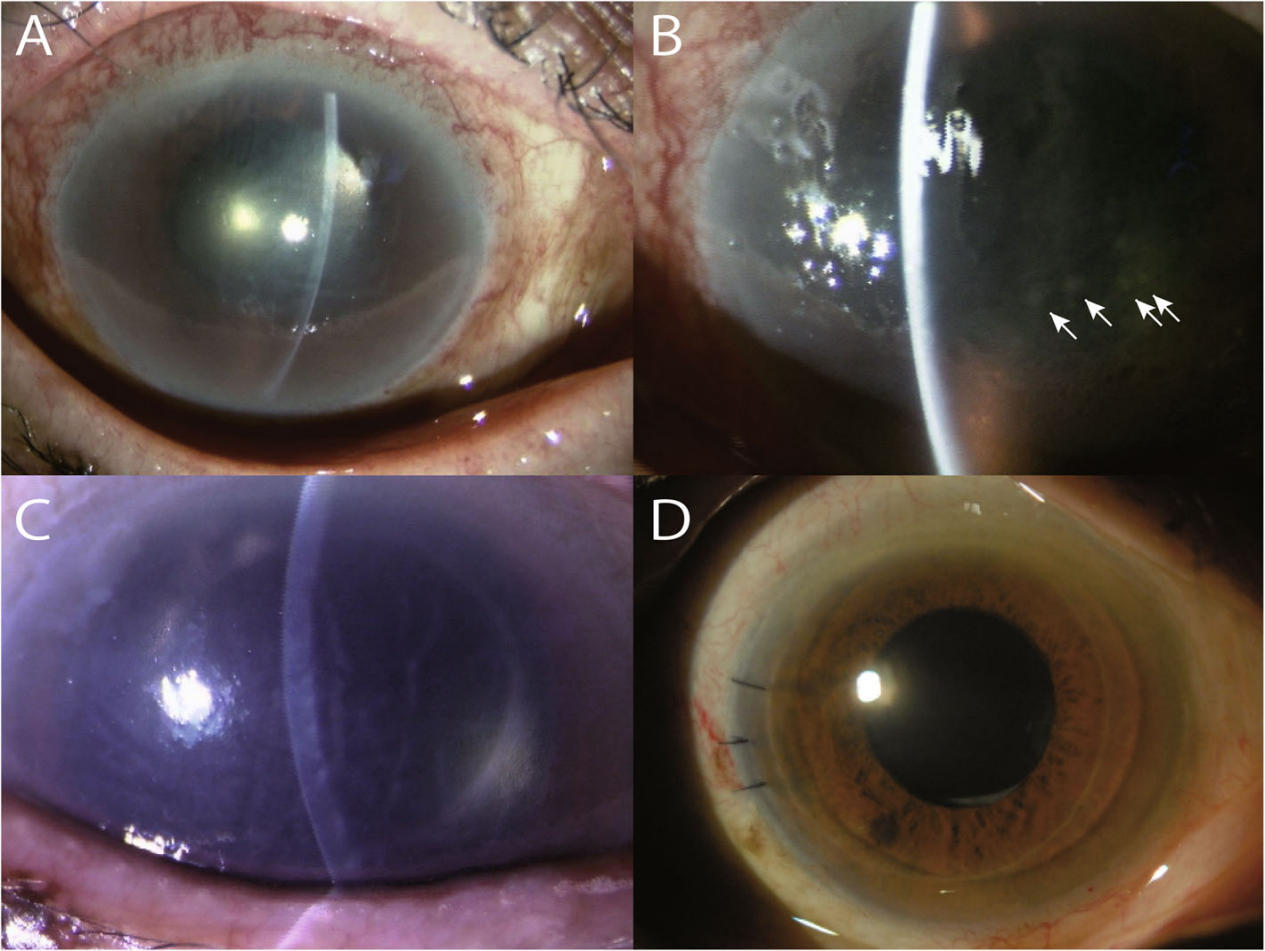
Anterior segment photographs of the eye of an 80-year-old woman with cytomegalovirus corneal endotheliitis. **a** At the time of the first visit, bullous keratopathy was observed, but inflammation was not observed. **b** At the time of relapse of inflammation, mutton fat keratic precipitates (KPs) were observed (arrows). **c** At the time just before Descemet-stripping automated endothelial keratoplasty (DSAEK), no inflammation was observed in the anterior chamber, and mutton fat KPs were not observed. **d** 6 months after DSAEK, corneal transparency was improved, and inflammation was not observed. KP, keratic precipitates; DSAEK, Descemet-stripping automated endothelial keratoplasty

An 80-year-old woman underwent bilateral cataract surgery at the age of 72 at a local clinic. Four months after the operation, iridocyclitis developed in the right eye, and was relieved by steroid instillation, though attacks occurred once or twice a year with elevated intraocular pressure (IOP). Corneal endothelial cells in the right eye gradually decreased, and the patient was referred to our department at the age of 80 for unexplained unilateral iridocyclitis and bullous keratopathy with hand motion vision in the right eye (Fig. 1a). She had significant corneal edema and inferior-localized pigmented KPs but no significant inflammation in the anterior chamber. IOP was 11 mmHg on betamethasone 0.1% four times a day (QID), bimatoprost 0.03% once per day (Lumigan; Senju Pharmaceutical Co., Ltd.), timolol 0.5%/brinzolamide 1.0% two times daily (BID) (Azorga®; Novartis), brimonidine 0.1% BID (Aiphagan; Senju Pharmaceutical Co., Ltd.), and ripasudil 0.4% BID (Glanatec®, KOWA Company Ltd.). Her central corneal thickness (CCT) was 745 μm and corneal endothelial density was uncountable. According to the interview at the first visit, there was no past history of infectious diseases including viruses.

An inflammation relapse occurred 8 months after the initial visit due to an interrupted dosage of medication. At that time, mutton fat KPs were observed (Fig. 1b) and IOP was elevated to 19.9 mmHg. Two weeks after resuming betamethasone and anti-glaucoma eye drops, inflammation was relieved and IOP was lowered to 12.9 mmHg.

We suspected CMV corneal endotheliitis from clinical observations; therefore, we acquired informed consent from the patient and discontinued betamethasone treatment in order to measure CMV DNA by real-time PCR of the aqueous humor with active inflammation. Two weeks after interruption of betamethasone, inflammation relapsed, mutton fat KPs were observed, and IOP was elevated to 25 mmHg. At that time, a quantitative real time PCR using the aqueous humor was performed, but DNA for CMV, herpes simplex virus (HSV) and varicella-zoster virus (VZV) were all negative. Clinical signs of inflammation resolved, and IOP was lowered to 14 mmHg a month after resuming betamethasone and anti-glaucoma eye drops. At the age of 81, 2 months later after the negative aqueous humor PCR, since KPs disappeared and no inflammation was observed with steroid instillation (Fig. 1c), DSAEK was performed in the right eye. After the corneal endothelial specimen during DSAEK was isolated and washed with intraocular infusion solution, DNA was extracted as manufacturer’s protocol by using QIAamp UCP Pathogen mini kit (Qiagen, Hilden, Germany) and real time PCR was performed. The results were positive for CMV-DNA at 6.0 × 10^2^ copies/ GAPDH 10^5^ copies [4], while those of HSV, VZV, and other five human herpes viruses were all negative. The patient was diagnosed with CMV corneal endotheliitis based on the PCR results and clinical settings. After confirming liver and kidney function with AST 24 mg/dl, ALT 21 mg/dl, creatinine 0.72 mg/dl blood and urea nitrogen 14.9 mg/dl, oral valganciclovir was administered at 900 mg BID for 2 weeks and then 450 mg BID for 6 weeks. She was also prescribed topical ganciclovir 1% six times a day for 2 months, which was slowly tapered to QID. No recurrence of iridocyclitis or corneal endotheliitis has been observed 6 months after DSAEK surgery (Fig. 1d). The patient’s corneal edema resolved (CCT was lowered to 607 μm), and best corrected visual acuity increased to 20/200.

## Discussion and conclusions

We demonstrate the successful utilization of a PCR test on removed corneal endothelial specimens during DSAEK to diagnose CMV corneal endotheliitis. Indeed, the case presented herein underscores that removed corneal tissue should be tested, even if the PCR using aqueous humor during inflammation does not detect CMV-DNA. In the present case, PCR test of the anterior aqueous humor during inflammation in the right eye did not detect CMV-DNA, but the subsequent PCR test of the corneal endothelial specimen removed during DSAEK did detect CMV-DNA even when inflammation had subsided.

At the time of the surgery, we conducted PCR only for corneal endothelial specimen but not for aqueous humor, because anterior segment inflammation has not been observed during the 2 months after confirming negative aqueous humor PCR. It would be interesting to confirm if the PCR results of the aqueous humor and the corneal removed tissue at the time of the surgery match.

As Ang et al. reported [5], CMV-positive corneal endotheliitis eyes showed higher recurrence rate within a year after corneal transplantation, as compared to CMV-negative eyes (60% vs 7.4% respectively). Since prophylactic use of topical ganciclovir was reported to be effective to prevent CMV recurrence [6], detections of potential CMV infection can be used to judge the initiation of prophylactic treatment. Therefore, considering the possibility of viral infection for recurrent iridocyclitis/corneal endotheliitis even if the aqueous humor PCR during inflammation cannot detect CMV-DNA, PCR of corneal endothelial specimen collected at the time of corneal transplantation is considered worthwhile.

In a recent report, clinical features of CMV-related anterior uveitis were reported to be variable although ethnic differences had not been observed. A prompt anterior tapping was recommended to manage the disease; however, it was important to consider that aqueous humor PCR examination could have had false negative results [7]. The aqueous humor PCR in this case did not detect CMV-DNA at the time of inflammation, possibly due to a small number of cells in the aqueous humor, which could be considered as a cause of the false negative result.

In histopathologic analyses of CMV corneal endotheliitis, previous reports revealed an owl’s eye appearance, a characteristic of CMV-infected cells, observed by confocal microscopy, and these findings suggest the direct infection of corneal endothelial cells with CMV [1, 8]. Although more cases need to be investigated, it is reasonable to assume that the corneal endothelial specimen has more sensitivity and a lower false-negative rate in PCR testing than the aqueous humor sample. Therefore, PCR testing for a corneal endothelial specimen removed during corneal transplantation could reveal potential CMV infection in some patients who tested false-negative with aqueous humor PCR.

In conclusion, we observed a case of CMV endotheliitis that was diagnosed by PCR of the removed Descemet’s membrane and endothelium during DSAEK surgery, even though the result of aqueous humor PCR was negative. This case suggests testing the removed endothelial tissue during DSAEK surgery using PCR to diagnose CMV endotheliitis for suspected but undiagnosed cases with the negative results of aqueous humor PCR.

## Data Availability

All the data supporting the findings is contained within the manuscript.

## Abbreviations

BID: Two times daily
CCT: Central corneal thickness
CMV: Cytomegalovirus
DSAEK: Descemet-stripping automated endothelial keratoplasty
HSV: Herpes simplex virus
IOP: Intraocular pressure
KP: Keratic precipitate
PCR: Polymerase chain reaction
QID: Four times a day
VZV: Varicella-zoster virus
